# Properties of the Force Exerted by Filopodia and Lamellipodia and the Involvement of Cytoskeletal Components

**DOI:** 10.1371/journal.pone.0001072

**Published:** 2007-10-24

**Authors:** Dan Cojoc, Francesco Difato, Enrico Ferrari, Rajesh B. Shahapure, Jummi Laishram, Massimo Righi, Enzo M. Di Fabrizio, Vincent Torre

**Affiliations:** 1 Consiglio Nazionale delle Ricerche (CNR)-Istituto Nazionale per la Fisica della Materia (INFM), Laboratorio Nazionale Tecnologie Avanzate E Nanoscienza (TASC), Area Science Park Basovizza, Trieste, Italy; 2 International School for Advanced Studies (SISSA-ISAS), Trieste, Italy; 3 Università Magna Graecia di Catanzaro, Campus Germaneto, Catanzaro, Italy; 4 Centro per la Biomedicina Molecolare (CBM), LANADA Laboratory, Trieste, Italy; 5 Italian Institute of Technology, International School for Advanced Studies (ISAS) Unit, Italy; Medical College of Georgia, United States of America

## Abstract

During neuronal differentiation, lamellipodia and filopodia explore the environment in search for the correct path to the axon's final destination. Although the motion of lamellipodia and filopodia has been characterized to an extent, little is known about the force they exert. In this study, we used optical tweezers to measure the force exerted by filopodia and lamellipodia with a millisecond temporal resolution. We found that a single filopodium exerts a force not exceeding 3 pN, whereas lamellipodia can exert a force up to 20 pN. Using metabolic inhibitors, we showed that no force is produced in the absence of actin polymerization and that development of forces larger than 3 pN requires microtubule polymerization. These results show that actin polymerization is necessary for force production and demonstrate that not only do neurons process information, but they also act on their environment exerting forces varying from tenths pN to tens of pN.

## Introduction

During morphogenesis, neuronal precursor cells migrate from the zone where they are born to their final destination, which, in some cases, is at a distance of several millimeters[1], [2]. After reaching their destination, neurons must establish appropriate synaptic connections by sending out from their soma projections called neurites. The motion of neurites is guided by growth cones located at their tips[3], [4]. Growth cones contain a variety of chemical and mechanical receptors and sophisticated biochemical machinery that couples these receptors to the cytoskeleton[5]–[7]. Extruding from the tip of the growth cone are highly motile structures called filopodia and lamellipodia that are used to explore and probe the environment[3], [6]. All these complex events, which are at the basis of neuronal development and differentiation, involve cell motility requiring a precise control of cellular and molecular motors. The motion of these structures has been analyzed and characterized to some extent by time-lapse microscopy[8]–[12].However, little is known about how neurons use these structures to sense the mechanical properties of their environment and about what range of forces these structures exert during their exploratory motion.

Analysis of the forces exerted by neurons has been limited to theoretical considerations; experimental analysis has been limited to samples of isolated filaments[13]–[17]or migrating cells[18], [19]. Measured forces range from 1 or 2 pN in isolated actin filaments and microtubules to 1 nN in migrating keratocytes. Quantitative characterization of the force exerted by lamellipodia and filopodia during neuronal differentiation could help to elucidate how neurons sense the environment and process mechanical information. Precise description of the mechanical and dynamic events that occur during neuronal differentiation and migration would provide new insights regarding the molecular events controlling these biological functions. In addition, it would offer a more precise way for evaluating the role of molecular motors in cell motility under physiological conditions and in neurodegenerative disease.

In this study, we used optical tweezers[20]–[22] to measure the force exerted by filopodia and lamellipodia during neuronal differentiation. Unlike other force measurement methods, optical tweezers are non-invasive and provide direct high temporal resolution for position detection (<10 nm) and force measurement (<1 pN), highly relevant in biological systems[21]. We found that a single filopodium exerts a force not exceeding 3 pN. In contrast, lamellipodia exert forces of 20 pN or more lasting less than 1 s to approximately 30 s. Treatment of growth cones with the selective myosin light chain kinase (MLCK) inhibitor ML-7[23] or the microtubule depolymerizing agent nocodazole[24] drastically reduced the motion and force exerted by lamellipodia, while filopodia continued to move and exert forces up to 3 pN. Growth cones treated with the actin depolymerizing agent latrunculin A[24] did not exert any detectable force. These findings suggest that no force can be produced in the absence of actin polymerization and that development of forces larger than 3 pN requires microtubule polymerization. This study shows that not only do neurons process information, but also they act on the environment, exerting forces varying 1 to 2 orders of magnitude.

## Results

During neuronal differentiation and development, the growth cone of each neurite extends its filopodia and lamellipodia to explore the chemical nature of the environment and to probe the rigidity and composition of the extracellular matrix[23]. Under these circumstances, cell motility is strictly linked to the generation of forces. Therefore, we used optical tweezers[20]–[22] to measure the force exerted by the growth cones of differentiating neurons.

### Force exerted by growth cones of differentiating neurons

Neurons from dorsal root ganglia (DRG) were isolated from P10-P12 rats and plated on poly-L-lysine-coated glass coverslips and positioned on the stage of an inverted microscope that was used for imaging and measurement of forces (see Methods). After incubation for 24 to 48 h, neurites could be seen emerging from the DRG soma. Their motion was analyzed with time-lapse differential interference contrast microscopy (Movie S1). Filopodia and lamellipodia moved rapidly, exploring the three-dimensional space in all directions, with velocities of up to 1.2 µm s-1 and reaching heights up to 1–3 µm.

**Figure 1:**
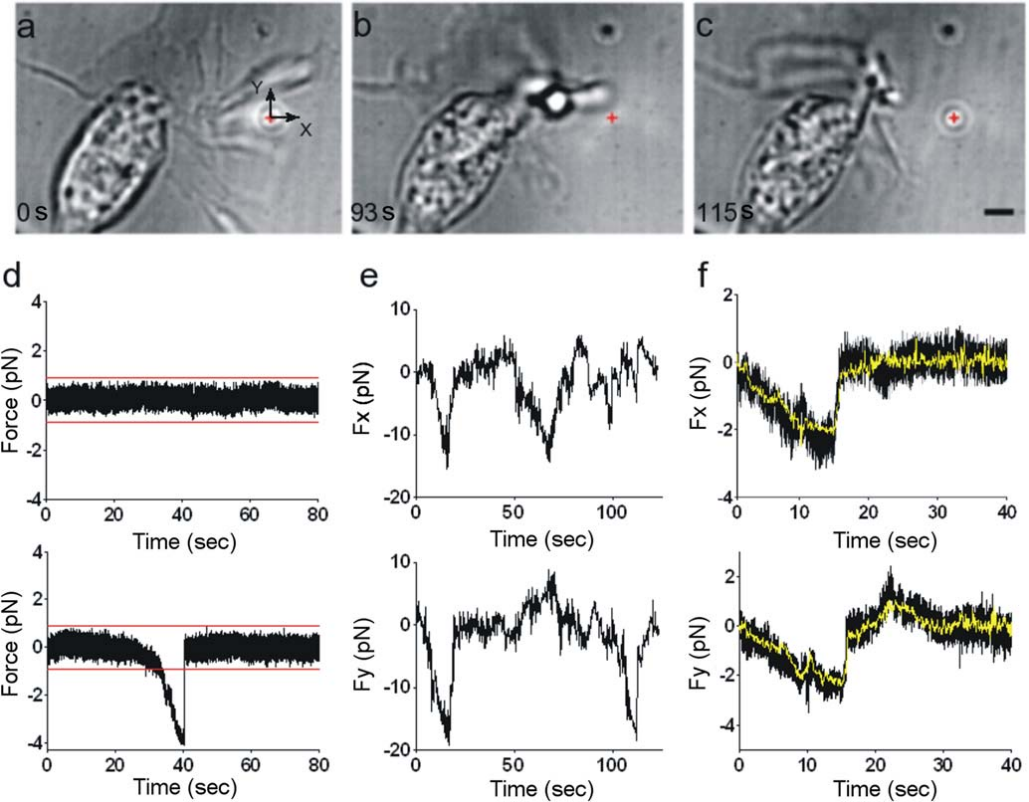
Collisions between a growth cone and a trapped bead. (a–c) A growth cone displacing a bead from the optical trap. The red cross indicates the bead's equilibrium position inside the optical trap. Scale bar, 2 µm. (d) Example of a force component obtained with QPD when the bead was distant from the growth cone (upper trace) and when the bead was in contact with the growth cone (lower trace). Red lines are drawn 5 σ from the 0 mark. σ, s.d. of force fluctuations. When the QPD trace crossed the red lines for at least 100 ms and a lamellipodium or filopodium was seen hitting the bead, a reliable collision was detected. (e) Example of F_x_ and F_y_ during repetitive collisions between a moving lamellipodium and a trapped bead. Trap stiffness was 0.05 pN nm^−1^. (f) Comparison of F_x_ and F_y_ determined with a QPD (black traces) and video tracking (yellow traces).

Silica beads 1 µm in diameter were functionalized with amino groups to reduce sticking and trapped with 1064-nm infrared optical tweezers (laser power between 8 and 44 mW) close to the growth cones of the differentiating neurites (Fig. 1a and Movie S2). We verified that 50 mW laser power reaching the specimen plane and focused on the growth cone did not affect its motion for at least 1 h. Often we observed both lateral and axial displacement of the trapped bead by a growth cone. In several experiments, the growth cone moved the bead as much as 2–3 microns from its equilibrium position inside the trap (Fig. 1b). After the collision, the bead did not remain attached to the growth cone and could return to its original position in the trap (Fig. 1c). We measured the lateral force exerted by the growth cone Fneu = (Fx, Fy) by following the bead position with a quadrant photo diode (QPD)[22] and video tracking[25] (see Methods). When the bead was far from the growth cone, QPD recordings of Fx and Fy were quiet, with a s.d. σ of approximately 0.18 pN (Fig. 1d, upper trace ), but when the bead was moved close to the growth cone, collisions producing a force greater than 5 σ were observed (Fig. 1d, lower trace). On several occasions, Fx and Fy increased within 1–10 s, reaching values of 20 pN (Fig. 1e), and when the growth cone stopped pushing, the bead rapidly returned to its equilibrium position, often in less than 1 ms.

The presence of floating debris and wandering filopodia near the bead could affect the light pattern impinging on the QPD. Therefore, a collision was considered reliable when the bead displacement obtained with the QPD and video tracking were in agreement (black and yellow traces, respectively, in Fig. 1f) and the presence of a colliding filopodium or lamellipodium was verified by visual inspection of the movie. We analyzed collisions between growth cones and trapped beads in more than 200 experiments. Each experiment lasted 2 min, and in many experiments there were several collisions that could be used for statistical analysis (see Methods). These collisions produced maximal forces ranging from less than 1 pN to at least 20 pN, with a maximal rate of increase of 10 pN s-1. They lasted for less than 1 s to approximately 60 s. Typically, larger forces were observed during longer lasting collisions. As these forces extended over a wide range of intensities and durations, we took the further step of characterizing the force developed by each major component of growth cones, filopodia and lamellipodia.

### Force exerted by filopodia

**Figure 2:**
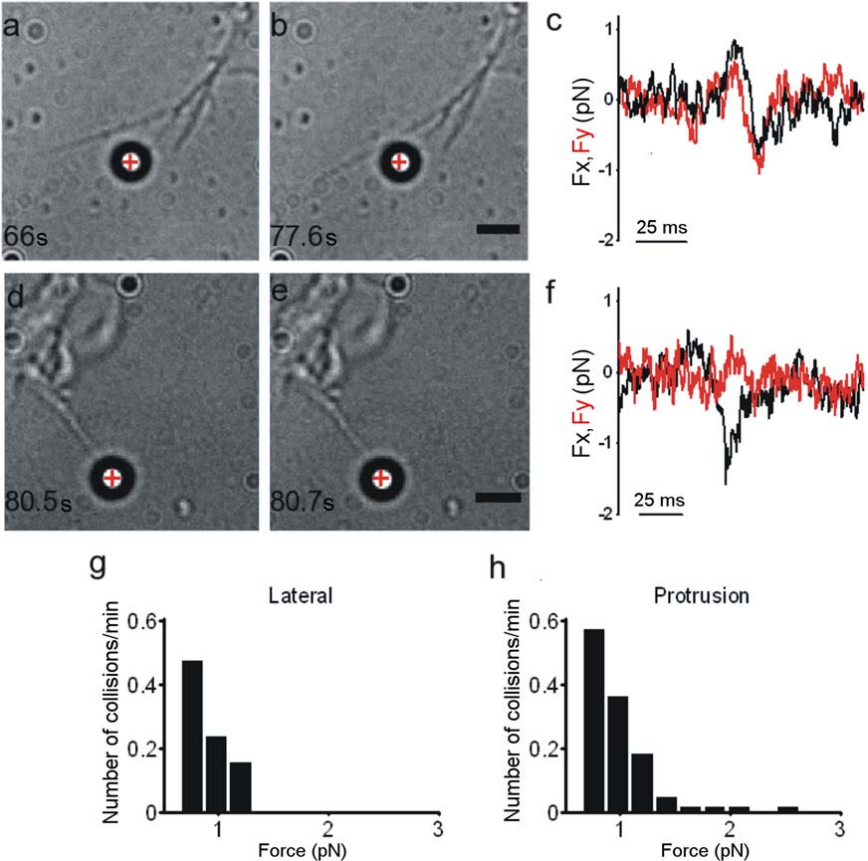
Force exerted by Filopodia. (a–b) Lateral collision between a filopodium and a trapped bead. Trap stiffness was 0.008 pN nm^−1^. The red cross indicates the bead's equilibrium position inside the optical trap. (c) F_x _and F_y_ from the QPD during the lateral collision shown in (a–b). (d–e) Collision between a protruding filopodium and a trapped bead. (f) F_x_ and F_y_ from the QPD during the filopodial protrusion shown in (d–e). Trap stiffness was 0.008 pN nm^−1^. (g–h) Histograms of forces measured during lateral collisions and protrusions. Data were collected from 75 experiments, each lasting 2 min. Scale bar, 2 µm.

Filopodia have an elongated and well defined shape with diameters varying from 100 to 500 nm and an average length of approximately 15 µm[26]. Filopodia can exert force during both exploratory motion and growth. During their exploratory motion often filopodia pivot and push beads aside, possibly as a consequence of shearing movements of the lamellipodial actin network where the filopodial shaft emerges. We refer to the first case as lateral collisions and to the latter case, where the filopodium pushes the bead, as protrusion. An isolated filopodium, after wandering around the bead (Fig. 2a), sometimes collided with it (Fig. 2b and Movie S3), exerting a maximal force of up to 1 pN (Fig. 2c). The force measured during lateral collisions depends on the exact geometry of the collision: a moving filopodium can strike a trapped bead at its center or just lightly touch its surface. Results from 42 experiments show that filopodia never exerted a force larger than 2 pN (Fig. 2g), which is a reliable upper boundary for the maximal force exerted during a lateral collision. Some lateral collisions lasted less than 1 s, but on several occasions we observed filopodia pushing beads for 15 s.

The force exerted by a filopodium is generated by its elastic properties[19] and a variety of molecular processes[27], including polymerization of actin filaments[28], [29], which generates a protrusion force counterbalanced by the membrane resistance force[30]–[32], leading to a net force Ftip. To measure forces produced during protrusion, beads were trapped in front of filopodia tips (Fig. 2d). In 33 experiments, we observed protruding filopodia displacing beads, often repeatedly (Fig. 2e and Movie S4; see also Figure S1 and Movie S5). The measured force was approximately 1 pN, and it developed within 30 ms (Fig. 2f). Ftip did not exceed 3 pN (Fig. 2h). These collisions rarely lasted more than 30 s.

**Figure 3:**
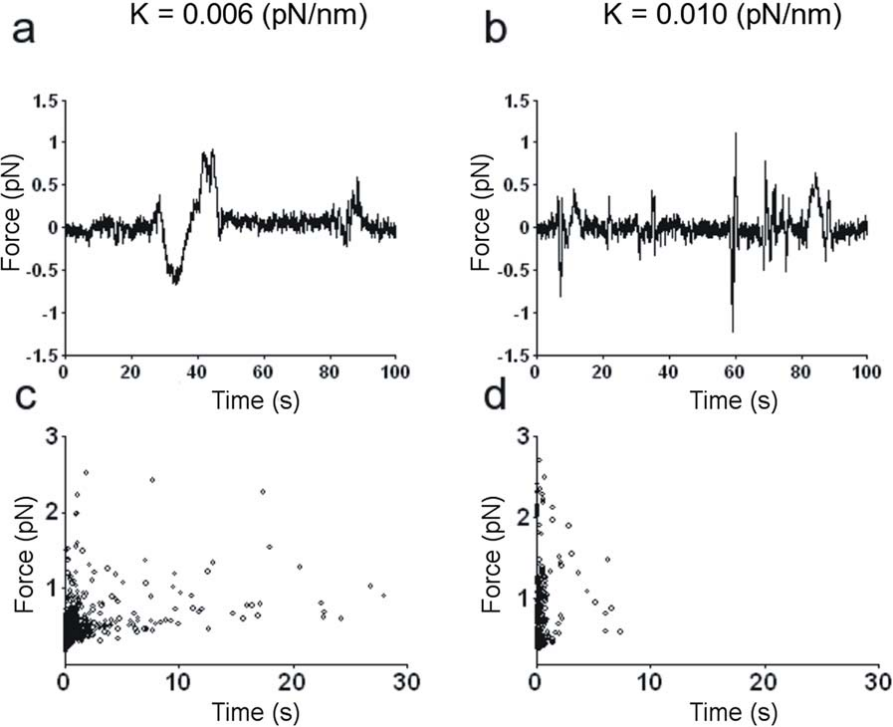
Effect of trap stiffness on force exerted. (a–b) F_x_ from the QPD during collisions between the same filopodium and the same bead trapped with a stiffness of 0.006 and 0.010 pN nm^−1^. Traces were filtered at 50 Hz and sub-sampled. (c–d) Scatter plot of force duration for collisions between filopodia and beads trapped with a stiffness of 0.006 and 0.010 pN nm^−1^. Data collected from 15 experiments at each stiffness.

When a filopodium collides with an encountered obstacle, it senses the object's chemical properties and also probes its mechanical resistance and size. Therefore, we investigated whether the force exerted by filopodia varies with the stiffness of the optical trap. We conducted several experiments in which we increased the trap stiffness from 0.006 pN nm-1 to 0.01 pN nm-1 and analyzed the collisions that occurred between the same growth cone and trapped beads. Under the two conditions of trap stiffness, collisions produced forces similar in magnitude (Fig. 3a, b), but collisions with beads trapped with a higher stiffness appeared to be shorter in duration. Data from 18 experiments show a similar distribution of measured forces under the two conditions but more frequent longer lasting collisions with the lower trap stiffness (Fig. 3c, d).

As shown in Figure 3, filopodia appeared to modulate their mechanical response by decreasing the duration of the collision when encountering a stiffer obstacle. Thus, they appear to be able to communicate the mechanical properties of the environment to the internal biochemical machinery that powers the cytoskeleton.

### Force exerted by lamellipodia

**Figure 4:**
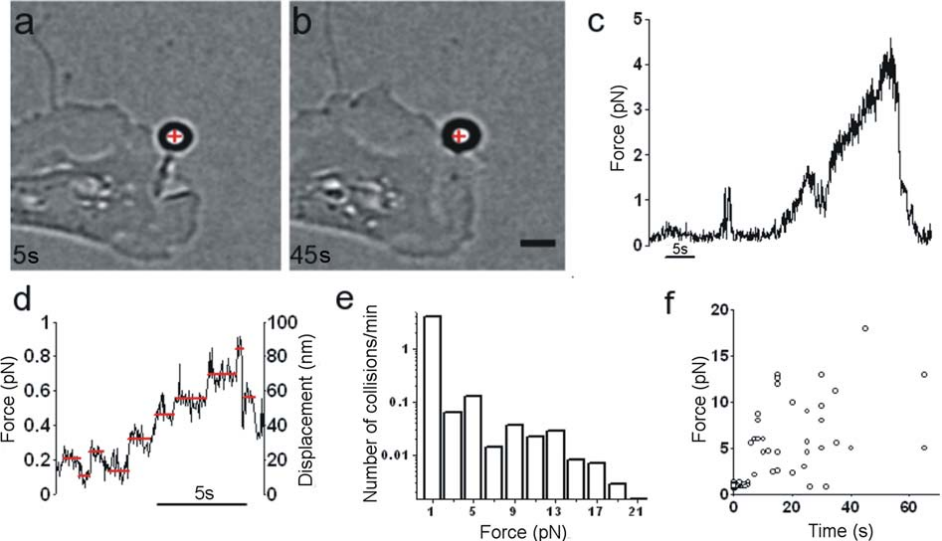
Force exerted by lamellipodia. (a–b) A lamellipodium growing and pushing a trapped bead. The red cross indicates the equilibrium position inside the optical trap. Scale bar, 2 µm. (c) F_neu _in the x,y plane obtained from a QPD recording. Trap stiffness was 0.009 pN nm^−1^. (d) The force exerted by a lamellipodium showing step-like jumps. Red lines, drawn by eye, indicate presumed discrete levels. The QPD recording was sub-sampled and filtered at 50 Hz. After low-pass filtering, the value of σ was reduced to 0.05 pN. Trap stiffness was 0.01 pN nm^−1^. (e) Histogram of forces measured during collisions between lamellipodia and trapped beads. Data reflect 65 experiments, each lasting 2 min. (f) Scatter plot of force duration for the collisions shown in (e).

We often observed that a lamellipodium repeatedly pushes a trapped bead (Fig. 4a,b and Movie S6), exerting a force of 3–4 pN (Fig. 4c). Lamellipodia could displace beads from the trap when the maximum trapping force was 20 pN. In 6 experiments, we observed lamellipodium increasing the exerted force in well resolved steps of approximately 0.2 pN, corresponding to displacements of approximately 18 nm (Fig. 4d). These steps have properties very similar to those observed during microtubule assembly, where discrete jumps of approximately 20–30 nm are observed[16]. In 65 experiments, lamellipodia exerted a force ranging from less than 1 pN to at least 20 pN, with a variable duration (Fig. 4e, f).

**Figure 5:**
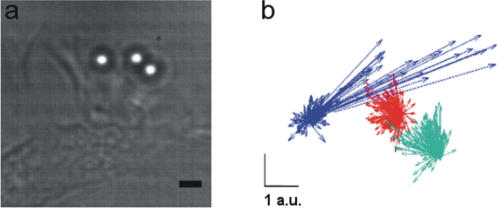
Force field exerted by lamellipodia. (a) A lamellipodium colliding with three trapped beads. (b) Direction and amplitude (in arbitrary units, a.u.) of forces exerted on the three beads. Superposition of bead displacements was obtained by video tracking at 5 Hz from a 4-min recording. Scale bar, 2 µm.

An isolated filopodium is complex from a molecular point of view, but it has a defined structure, and the force it exerts is well localized in space. In contrast, lamellipodia have a more differentiated structure and are thought to exert a force with variable direction in space. Therefore, we attempted to characterize the force field generated by lamellipodia by trapping multiple beads in front of a lamellipodium. Traps were separated by 3–6 µm and located on the same plane. In several experiments, 3 beads were displaced simultaneously by the lamellipodium (Fig. 5a), and we determined their trajectory with video imaging. The direction of forces at the three locations changed during the experiment and could span a large fraction of the free space surrounding the moving lamellipodium (Fig. 5b).

The force simultaneously exerted at the two locations separated by 3 µm was sometimes in opposite directions, and often the direction of force exerted at one location reversed within 10 s. This confirms that the force field generated by a lamellipodium is complex and dynamic over a short time scale.

### Effects of metabolic inhibitors on force exertion

**Figure 6:**
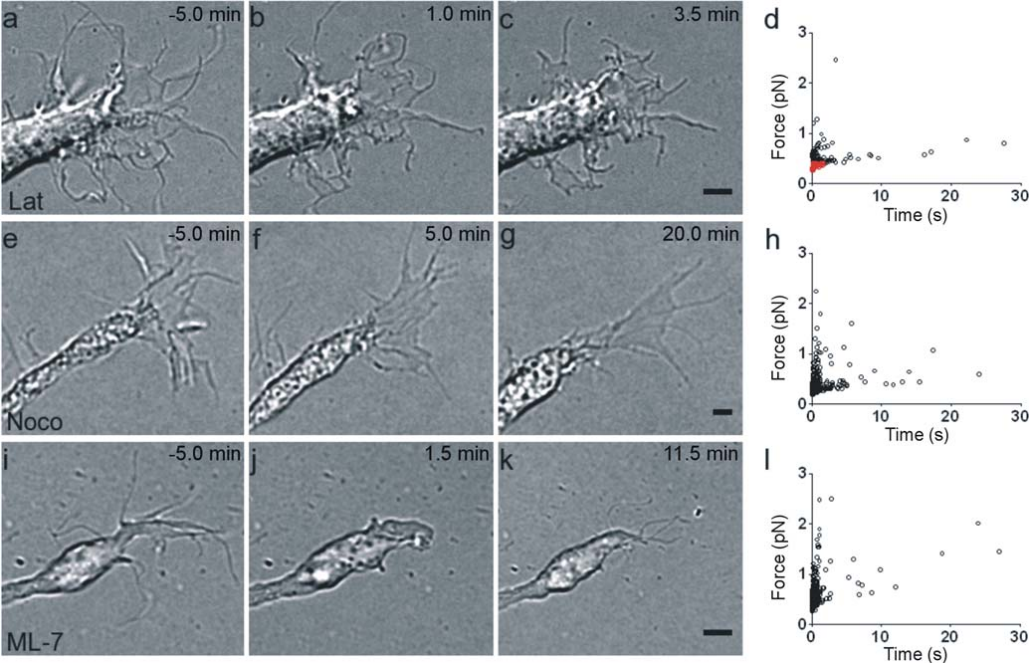
Effect of inhibitors on force exertion. A growth cone before (a) and after (b–c) application of 100 nM latrunculin A. No motion was observed after 3.5 min of exposure. (d) Scatterplot of force duration for collisions after application of 50 nM (black symbols) and 100 nM (red symbols) latrunculin A. A growth cone is shown before (e) and after (f–g) application of 50 nM nocodazole. The growth cone retracted, but filopodia continued to move for at least 30 min after drug exposure. (h) Scatterplot of force duration for collisions after application of 50 nM nocodazole. A growth cone is shown before (i) and after (j–k) application of 4 µM ML-7. Filopodia quickly retracted but then regrew and moved for at least 20 min after drug application. (l) Scatterplot of force duration for collisions after application of 4 µM ML-7. Scale bars, 2 µm. Drugs were added at time 0.

In order to identify the molecular mechanisms of force production, we analyzed the effect of metabolic inhibitors at concentrations known to be effective[23], [24]. Within 5 min after addition of 50 nM latrunculin A, an inhibitor of actin polymerization[24], the exploratory motion of growth cones was drastically reduced; under this condition, the force exerted by filopodia and lamellipodia did not exceed 3 or 4 pN, and collisions were shorter (black symbols in Fig. 6d). When the concentration of latrunculin A was increased to 100 nM, moving filopodia collapsed (Fig. 6a–c), and no detectable motion or force was observed in filopodia or lamellipodia (red symbols in Fig. 6d). In contrast, addition of 50 nM nocodazole, an inhibitor of microtubule polymerization[24], had a more specific effect. It reduced the motion of lamellipodia but not of filopodia, which continued to move (Fig. 6e–g), exerting a force of up to 3 pN (Fig. 6e). Upon addition of 4 µM of the myosin II inhibitor ML-7[23], a fast retraction of moving filopodia was observed (Fig. 6i–j), but within 2–5 min new filopodia emerged from the growth cone (Fig. 6k), which exerted a force in the pN range (Fig. 6l).

These results suggest the existence of two distinct but coupled molecular motors within growth cones. Actin polymerization seems necessary for the development of any significant motion and force in filopodia and lamellipodia. Microtubule polymerization is not essential for filopodia motion or for the generation of weak forces, but it is necessary for lamellipodia motion and generation of forces larger than 3 pN.

## Discussion

A typical growth cone can be divided into two regions: the central region and the peripheral region. The latter consists of filopodia and a lamellipodia[33]–[36]; the motion of these structures is a major component of neuronal differentiation. This is the first report of a quantitative determination with a millisecond temporal resolution of the force exerted by filopodia and lamellipodia in differentiating neurons. The force developed over time, with a maximal rate of increase of 10 pN s-1. Thin filopodia, during a protrusion or lateral collision (Fig. 2), exerted a force not exceeding 3 pN. In contrast, lamellipodia exerted a force of up to 20 pN and possibly more, which could increase in discrete steps of approximately 0.2 pN (Fig. 4d). These steps had properties very similar to those observed during the assembly of isolated microtubules[16]. The measured forces were smaller than forces involved in cellular traction force or measured in migrating keratocytes[15], [18]. Measured forces here reported, may not fully represent the ability that lamellipodia have because, at least in some cases, only a fraction of the forces exerted is picked up by the beads and therefore the value of 20 pN here reported for lamellipodia is the maximal force that was measured. Indeed we expect lamellipodia to exert larger forces, possibly up to hundreds of pN, as in migrating epithelial cells[37]. The diameter of filopodia tips is approximately 100 nm, i.e. 10 times smaller than the diameter of the used beads, therefore the maximal force measured for filopodia is expected to be a reliable estimate of the force exerted by these structures.

Force measurements with optical tweezers require test beads to be in the harmonic potential well of the trapping optical force and to be displaced from its equilibrium position inside the well only by the force to be measured. When adhesion forces between the bead and the growth cone and/or between the bead and the substrate become dominant, the stiffness of the optical trap is profoundly modified and it is impossible to obtain an accurate force measurement. Therefore, it is necessary to place the bead at 1 micron or so from the substrate where neurons are growing. As exploring filopodia and lamellipodia lift up from the substrate, forces can be reliably measured under these conditions.

Filopodia are composed of bundles of actin filaments and occasional microtubules [5], [24]. We observed that the force exerted by a protruding filopodium is in the pN range, not exceeding 3 pN. Its amplitude is of the same order as that measured during polymerization of actin filaments[14], [17] and microtubules[16]. This similarity implies that the protrusion force generated by polymerization is minimally counterbalanced by the membrane surrounding actin bundles and microtubules, indicating that the membrane at the filopodia tips has a low stiffness[38], [39].

Simple mechanical considerations show that the force exerted by a wandering filopodium during a lateral collision (Fig. 2a–c) can be accounted for by the elastic force expected from its flexural rigidity[19], [40] and its bending or buckling. No additional contribution from other force-generating mechanisms is required. The exact mechanisms causing filopodia to bend and/or buckle are not understood. Thermal fluctuations certainly provide a constant driving force, but a variety of other motor proteins[19], [29,29] present in the growth cone could intervene, although their relative contribution is still unknown. Indeed, inhibition of myosin II and microtubule polymerization blocked lamellipodia motion and drastically reduced the force produced by growth cones (Fig. 6), while filopodia continued to move and were able to exert forces in the pN range. In contrast, with blockade of actin polymerization, filopodia and lamellipodia produced no measurable forces. Thus, in the absence of actin polymerization, growth cones cannot exert any force, and microtubule polymerization is necessary for development of forces exceeding 3 pN. Therefore, actin filaments and microtubules cooperate and interact in a complex way so as to generate a wide range of forces.

The motion of filopodia and lamellipodia seems to follow stereotyped patterns wherein the stiffness of an obstacle is first probed. Often, an isolated filopodium changed its direction of growth after colliding with a trapped bead. In contrast, lamellipodia could remove an obstacle, often by growing underneath it and lifting it. Exploring filopodia exerted forces in the pN range, whereas migrating cells exert forces in the nN range[18]. A migrating neuron must be able to displace large obstacles; hence, it uses large forces. Filopodia gently explore their environment using only weak forces, and lamellipodia can exert a larger force opening the way for the growth cone. Thus, not only do neurons process information but they are also able to mechanically modify their environment by selecting forces varying from less than 1 pN to 1 nN[18]. Indeed, differentiating neurons sense the mechanical and chemical properties of barriers in front of their neurites and appear to have smart molecular motor planning, which guides and modifies the ultimate direction taken by neurites in the developing nervous system. Notably, these capabilities are in sharp contrast with metal and/or silicon components used for commercial information processing, which lack motility and motor planning.

## Materials and Methods

Rats (P10−12) were anesthetized with CO2 and sacrificed by decapitation in accordance with the Italian Animal Welfare Act. DRGs were incubated with trypsin (0.5 mg/ml), collagenase (1 mg/ml), and DNase (0.1 mg/ml) in 5 ml Neurobasal medium in a shaking bath (37°C, 35–40 min). They were mechanically dissociated, centrifuged at 300 rpm, resuspended in culture medium, and plated on poly-L-lysine-coated (0.5 ug/ml) coverslips. Cells were incubated for 24 to 48 h, and nerve growth factor (50 ng/ml; Alomone, Israel) was added before measurements were obtained.

The optical tweezers setup was built as previously described.[41] The dish containing the differentiating neurons and the beads (PSI-1.0NH2; G.Kisker GbR, Steinfurt, Germany) was placed on a microscope stage, which could be moved by a 3-axis piezoelectric nanocube (17 MAX 301; Melles Griot Inc., USA). The temperature of the dish was maintained at 37°C using a Peltier device. Bead position was determined in the x,y plane with an accuracy of 10 nm, using back focal plane (BFP) detection which relies on the interference between forward scattered light from the bead and unscattered light[22], [42]. The BFP of the condenser was imaged onto a QPD, and the light was converted to differential outputs digitized at 4 kHz and low-pass filtered at 2 kHz. The bead displacement d = (dx,dy) from the equilibrium position inside the optical trap was also determined by video tracking using correlation methods with sub-pixel resolution. The lateral trap stiffness κx,y = (kx,ky) and the detector sensitivity were calibrated using the power spectrum method[22], with voltage signals filtered and digitized at 5 and 20 kHz, respectively. For multiple trapping experiments, computer-generated diffractive optical elements were projected onto the liquid crystal display of the phase-programmable modulator (PPM X8267-11; Hamamatsu Photonics, Japan)[41], [43] in order to generate multiple spots in the specimen with a Gaussian intensity profile. For experiments where a single Gaussian beam was required, the PPM was switched off. In multiple trapping experiments, only the direction of the force was determined but not its amplitude.

For statistical analysis, QPD traces were low-pass filtered at 50 Hz. Collisions selected for statistical analysis had to satisfy three criteria: 1) maximal amplitude larger than 5σ, 2) duration longer than 100 ms, and 3) presence of a colliding filopodium or lamellipodium in contact with a bead verified by visual inspection of the movie. The collision duration was calculated as the interval between two consecutive crossings of 5σ. The force exerted by the neurite Fneu was calculated as -Ftrap. When the displacement of the bead from its equilibrium position inside the trap was less than 400 nm, Ftrap = (Fx, Fy) was calculated as Fx = dxkx and Fy = dyky[22]. When the bead was also moved along the vertical axis, the lateral displacement measured with the QPD was compared with data obtained from video tracking; the data were discarded if lateral displacements measured with the two methods differed by more than 50%. The axial force along the z axis was not measured.

## Supporting Information

Figure S1 (a–b) Another example of a collision between a protruding filopodium and a trapped bead . The filopodium grows and hits the trapped bead. Trap stiffness was 0.006 pN/nm. c: Fy from the QPD during the protrusion lateral of a–b. Scale bar, 2 µm. Numbers in the lower right corner indicate time in seconds.
(3.29 MB TIF)

Movie S1 Movie of the motion of a growth cone imaged with time-lapse differential interference contrast (DIC) microscopy on the surface of the coverslip where the growth cone is located and at three focal planes 1, 2 and 3 µm above the coverslip. The four planes were scanned every 5 seconds. Filopodia are often seen in focus at 2 and 3 µm from the coverslip. (Acquisition rate: 5Hz; Scale bar, 2 µm). Numbers in the upper right corner indicate time in seconds.
(1.97 MB MOV)

Movie S2 Movie of the collision between the growth cone and a trapped bead shown in Fig.1a–c. The trap stiffness was 0.02 pN/nm. The time of image acquisition is indicated in the corresponding frame (Acquisition rate: 5Hz; Scale bar, 2 µm). Numbers in the upper right corner indicate time in seconds.
(4.95 MB MOV)

Movie S3 Movie of the lateral collision between the filopodium and a trapped bead shown in Fig.2a–b. The trap stiffness was 0.006 pN/nm. (Acquisition rate: 20Hz; Scale bar, 2 µm). Numbers in the upper right corner indicate time in seconds.
(4.07 MB MOV)

Movie S4 Movie of the collision between the protruding filopodium and a trapped bead shown in Fig.2d–e. The trap stiffness was 0.006 pN/nm. (Acquisition rate: 20Hz; Scale bar, 2 µm). Numbers in the upper right corner indicate time in seconds.
(4.45 MB MOV)

Movie S5 Movie of the collision between the protruding filopodium and a trapped bead shown in Supplementary Figure 1a–b. The trap stiffness was 0.006 pN/nm. (Acquisition rate: 20Hz; Scale bar, 2 µm). Numbers in upper right corner indicate time in seconds.
(3.60 MB MOV)

Movie S6 Movie of the collision between the lamellipodium and a trapped bead shown in Fig.4a–b. The trap stiffness was 0.02 pN/nm. (Acquisition rate 20Hz; Scale bar, 2 µm). Numbers in upper right corner indicate time in seconds.
(3.20 MB MOV)
