# Supplementary figures and images for: Properties of the Force Exerted by Filopodia and Lamellipodia and the Involvement of Cytoskeletal Components
Source: PLoS One. 2007 Oct 24;2(10):e1072. doi: 10.1371/journal.pone.0001072 (PMC2034605; doi:10.1371/journal.pone.0001072)

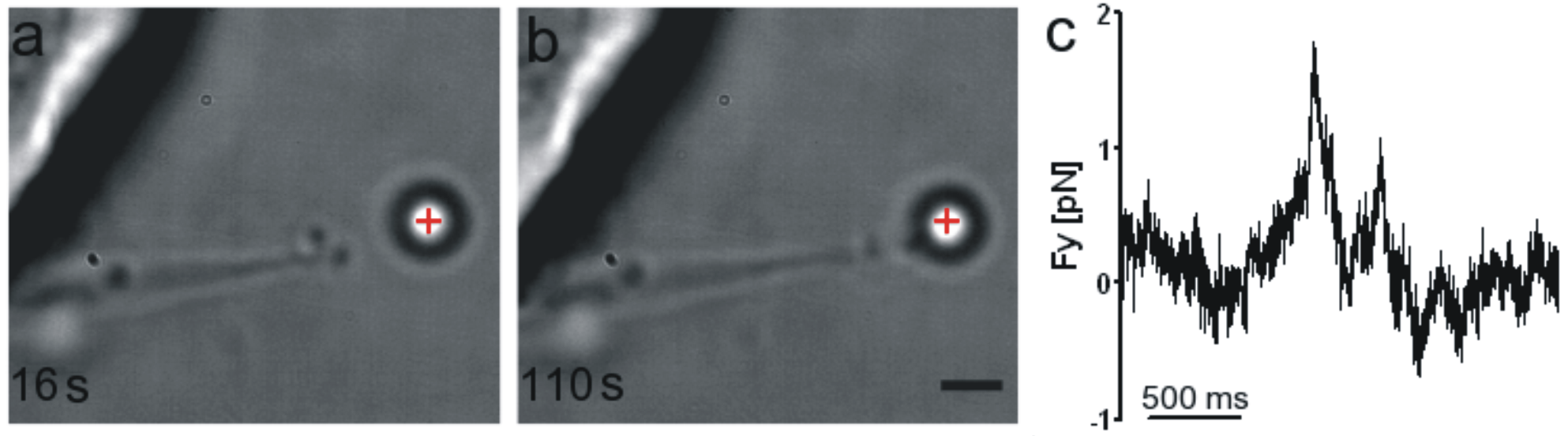

Supplement: Figure S1 — (a–b) Another example of a collision between a protruding filopodium and a trapped bead . The filopodium grows and hits the trapped bead. Trap stiffness was 0.006 pN/nm. c: Fy from the QPD during the protrusion lateral of a–b. Scale bar, 2 µm. Numbers in the lower right corner indicate time in seconds. (3.29 MB TIF) [file pone.0001072.s001.tif]
